# Metabolic adaptation and trophic strategies of soil bacteria—C1- metabolism and sulfur chemolithotrophy in *Starkeya novella*

**DOI:** 10.3389/fmicb.2013.00304

**Published:** 2013-10-17

**Authors:** Ulrike Kappler, Amanda S. Nouwens

**Affiliations:** School of Chemistry and Molecular Biosciences, The University of Queensland, St LuciaQLD, Australia

**Keywords:** carbon metabolism, methylotrophy, methanol, chemolithotrophy, thiosulfate, soil bacteria, *Starkeya novella*

## Abstract

The highly diverse and metabolically versatile microbial communities found in soil environments are major contributors to the global carbon, nitrogen, and sulfur cycles. We have used a combination of genome –based pathway analysis with proteomics and gene expression studies to investigate metabolic adaptation in a representative of these bacteria, *Starkeya novella*, which was originally isolated from agricultural soil. This bacterium was the first facultative sulfur chemolithoautotroph that was isolated and it is also able to grow with methanol and on over 39 substrates as a heterotroph. However, using glucose, fructose, methanol, thiosulfate as well as combinations of the carbon compounds with thiosulfate as growth substrates we have demonstrated here that contrary to the previous classification, *S. novella* is not a facultative sulfur chemolitho- and methylotroph, as the enzyme systems required for these two growth modes are always expressed at high levels. This is typical for key metabolic pathways. In addition enzymes for various pathways of carbon dioxide fixation were always expressed at high levels, even during heterotrophic growth on glucose or fructose, which suggests a role for these pathways beyond the generation of reduced carbon units for cell growth, possibly in redox balancing of metabolism. Our results then indicate that *S. novella*, a representative of the *Xanthobacteraceae* family of methylotrophic soil and freshwater dwelling bacteria, employs a mixotrophic growth strategy under all conditions tested here. As a result the contribution of this bacterium to either carbon sequestration or the release of climate active substances could vary very quickly, which has direct implications for the modeling of such processes if mixotrophy proves to be the main growth strategy for large populations of soil bacteria.

## Introduction

Agriculturally used soil surfaces account for approximately 37% of the Earth's total land area and thus the biogeochemical processes taking place in this vast ecosystem can affect the entire biosphere. Soil environments are also major contributors to global element cycles including the sulfur and carbon cycle both of which are important for agriculture (Kertesz et al., 2007; Kolb, 2009; Yuan et al., 2012).

As a result, the biological processes in soils contribute significantly to the release or consumption of a variety of climate active substances which include volatile sulfur compounds as well as methanol and carbon dioxide, both of which are important in the global carbon cycle and influence atmospheric chemistry (Kolb, 2009; Hunger et al., 2011). Methanol is known to contribute to ozone formation and is primarily produced and released by growing plants or the decay of plant materials. However, only a fraction of the methanol that is produced enters the atmosphere (~4.9 × 10^12^ mol year^−1^) while the rest is oxidized to carbon dioxide by methylo- and methanotrophic bacteria before being released (Kolb, 2009). The microbially mediated interconversion of compounds with a beneficial effect on atmospheric processes into ones with potentially detrimental effects has become a major focus of research into microbial metabolic activities as these processes can significantly influence the composition of atmospheric gases.

At present at least 56 bacterial species isolated from soils have been reported to be capable of degrading methanol, and the majority of these appear to be facultative methylotrophs (Kolb, 2009), suggesting that they are capable of switching to other growth modes which can include (chemolitho)autotrophy and heterotrophy using other reduced carbon compounds.

While historically bacteria have been classified as either autotrophs (i.e., fixing carbon dioxide) or heterotrophs based on their ability to grow on defined media in the laboratory, it has recently been proposed that these clear cut divisions may not accurately reflect natural processes (Eiler, 2006; Kolb, 2009). In nature bacteria likely encounter multiple energy and/or carbon sources at the same time, and in most cases these would not be present in high concentrations (Eiler, 2006; Kolber, 2007). Consequently, a “mixotrophic” growth strategy would increase the ability of bacteria to draw on multiple sources of energy rather than relying solely on hetero- or autotrophy at any given time (Eiler, 2006). However, little data on these processes are available at present despite their potential to affect microbial activities that contribute to carbon sequestration and/or the release of climate active substances.

In order to enhance understanding of trophic strategies in often highly versatile soil bacteria, we have investigated the differential expression of key metabolic pathway in the soil bacterium *Starkeya novella* (formerly *Thiobacillus novellus*) (Kelly et al., 2000) as a function of available growth substrates. *S. novella* was the first facultative sulfur oxidizing chemolithoautotroph to be isolated but is also capable of utilizing various C1 compounds, including methanol, for growth as well as at least 39 reduced carbon sources including sugars, amino sugars, amino acids, and organic acids (Starkey, 1935; Chandra and Shethna, 1977; Kelly et al., 2000; Kappler et al., 2012). This combination of metabolic traits should allow *S. novella* to contribute to both the biological sulfur and carbon cycles in various ways and depending on the prevailing growth mode its metabolic activities could either enhance carbon sequestration or the release of carbon dioxide. However, it has never been investigated if or how this bacterium makes use of the many possible growth modes that it is able to adopt. The only published studies of the consumption of sulfur compounds and sugars in *S. novella* yielded contradictory results reporting either simultaneous consumption or sequential use of energy sources (Lejohn et al., 1967; Leefeldt and Matin, 1980; Matin et al., 1980; Perez and Matin, 1980). The ability of *S. novella* to grow on C1 compounds also remains largely unexplored, with the only existing data being a report of robust growth on methanol and formate (Chandra and Shethna, 1977) and while *S.novella* sulfur metabolism was studied intensively in the 1960s and 70s (Aleem, 1965; Charles and Suzuki, 1966a,b; Oh and Suzuki, 1977a,b; Katayama Fujimura and Kuraishi, 1980), only some data that included molecular detail have been reported to date. In the last 15 years the presence of a *sox* gene cluster (*soxAX-soxYZBCDxF*) encoding a thiosulfate oxidizing multienzyme complex and a gene locus encoding a sulfite oxidizing enzyme (*sigEorf1-sorAB*) have been reported (Kappler et al., 2000, 2001, 2004).

Here we have used a combination of genomic, proteomic and gene expression data to identify key metabolic pathways involved in dissimilatory sulfur oxidation, utilization of C1 and other carbon compounds or growth in the presence of both types of energy sources with the aim of unraveling the relative activities of carbon sequestering and releasing pathways in *S. novella*.

## Methods

### Strains and growth conditions

*Starkeya novella* DSMZ506^T^ was routinely grown at 28°C on modified DSMZ69 medium as described elsewhere (Wilson and Kappler, 2009). The DSMZ69 medium base was supplemented with either 100 mM methanol (M, MeOH) or 40 mM thiosulfate (TS) or a combination of the two (TS/M, TS/MeOH). For strain maintenance DSMZ69 –TS agar plates supplemented with 40 μg/ml nalidixic acid were used. For proteomics experiments, liquid cultures were grown under microaerophilic conditions (100 ml medium in 250 ml shake flasks, 200 rpm, 28°C) to mid-late exponential growth phase, harvested by centrifugation and stored at −80°C until further use. For RNA isolation cultures were grown to mid-exponential growth phase before preservation with RNA protect bacteria reagent (Qiagen).

### Molecular methods

Standard methods were used throughout (Ausubel, 1995). Routine PCR used GoTaq Mastermix green (Promega) according to the manufacturer's instructions. Genomic DNA was isolated using the DNAZOL reagent (Life Technologies). Culture samples for RNA isolation (2 or 3 ml) were preserved in 1 vol of RNA protect bacteria reagent (Qiagen), RNA was isolated using the RNAspin mini Kit (GE Healthcare). RNA samples were tested for gDNA contamination using PCR, only samples that failed to produce a product after 34 cycles of amplification were used for cDNA synthesis. cDNA was prepared with Superscript III (Life Technologies) using 0.5 μ g of DNA-free RNA. Primer sets for use in qRT-PCR experiments (product size: 100 bp, annealing temperatures >60°C) (Table S1) were designed using Vector NTI Advance 11 (Life Technologies). qRT-PCR experiments were essentially performed as in (Kappler et al., 2005; Kappler and Nouwens, 2013) using the SYBR green Mastermix (Applied Biosystems) and 10 μ L reactions. Experiments were carried out at the University of Queensland SCMB realtime PCR facility using an epMotion workstation (Eppendorf) and an Applied Biosystems 7900 realtime PCR machine.

### Proteomics techniques

Cell pellets for use in MS/MS proteomics experiments were resuspended in 8 M urea, 50 mM ammonium bicarbonate pH 8.0 and disrupted using a French Pressure Cell (Aminco; 3 passes, 12000 psi). Samples were centrifuged (20,000 × *g*, 10 min) followed by determination of protein concentrations using the 2D Quant Kit (GE Healthcare). Between 1 and 2 mg of protein were then incubated with 5 mM DTT (30 min, 45°C) followed by treatment with 25 mM iodoacetamide (30 min, in the dark, RT). Samples were diluted 1 in 4 with 50 mM ammonium bicarbonate pH 8.0 before Trypsin Gold seq grade (Promega) was added in a 1:100 ratio. After 4 hours at 37°C the same amount of trypsin was added again and samples incubated overnight at 37°C.

Offline 2D LC-MS/MS analyses were used for shotgun proteomics as described in (Kappler and Nouwens, 2013). The equivalent of 500 μg of digested protein was diluted 1:1 with 5% ACN/0.1% TFA and desalted with a C18 Toptip (Glygen, USA). Desalting used 100% ACN to wet resin (3 × 150 μl), 5% ACN/0.1% TFA (3 × 150 μl) for tip equilibration and wash steps, and 80% ACN/0.1% TFA (2 × 150 μl) for elution. Eluted peptides were concentrated in a SpeedVac and resuspended in 0.5% acetic acid/2% ACN. Peptides (180 μg) were separated on an Agilent 1100 chromatography system using a Zorbax 300-SCX column (5 μm, 4.6 × 50 mm) (Agilent) at 0.5 ml/min (gradient: 0–5 min, 0% buffer B; 5–25 min, 0–50% buffer B; 25–27 min, 50–80% buffer B; 27–32 min, 80% buffer B; 32–34 min 80–0% buffer B, where buffer A = 0.5% acetic acid/2% ACN and buffer B = 0.5% acetic acid/2% ACN/250 mM ammonium acetate). Fractions (250 μl) were collected in a microtitre plate before pooling (final no. of pooled fractions: 10) and desalted using ZipTips (Millipore, Massachusetts, USA) followed by separation using reversed-phase chromatography on a Shimadzu Prominence nanoLC system. All fractions were analyzed in triplicate at a flow rate of 30 μl/min. Samples were first loaded on an Agilent C18 trap (0.3 × 5 mm, 5 μm) for 8 min, followed by separation on a Vydac Everest C18 (300 A, 5 μm, 150 mm × 150 μm) column at a flow rate of 1 μl/min. Peptides were separated on a gradient of 3–40% buffer B over 67 min followed by 40–98% buffer B over 3 min, where buffer A = 1% ACN/0.1% FA and buffer B = 80% ACN/0.1% FA was used. Eluted peptides were directly analyzed on a TripleTof 5600 instrument (ABSciex) using a Nanospray III interface. Gas and voltage settings were adjusted as required. MS-ToF scan across m/z 350–1800 was performed for 0.5 s followed by data-dependent acquisition of 20 peptides with intensity above 100 counts across m/z 40–1800 (0.05 s per spectra) using “high sensitivity” MS/MS and rolling collision energy.

MS data from triplicates of pooled samples were combined and searched using ProteinPilot v4.2 (ABSciex, Forster City CA) with the Paragon Algorithm using fasta formatted protein sequences for the finished *S. novella* genome obtained from JGI. Search parameters included trypsin as enzyme, iodoacetamide as cys-modification, emphasis on biological modifications and “thorough” search setting. Only proteins with a ProteinPilot confidence score of 95% or better (estimated global FDR 5% or lower) were accepted. Further data analysis used the integrated microbial genomes resource (IMG; img.jgi.doe.gov) (Markowitz et al., 2012).

### Bioinformatics

Analysis of the *S. novella* genome to identify pathways and enzyme systems was performed using the KEGG pathways database (www.genome.jp/kegg/pathway.html) (Kanehisa et al., 2012), comparative analyses used biocyc.org (*biocyc.org/*) (Caspi et al., 2012), and IMG (*img.jgi.doe.gov/*) (Markowitz et al., 2012). In some cases BLASTP (Altschul et al., 1997) was used to confirm the absence or presence of genes not identified in other database searches

## Results

**Figure 1:**
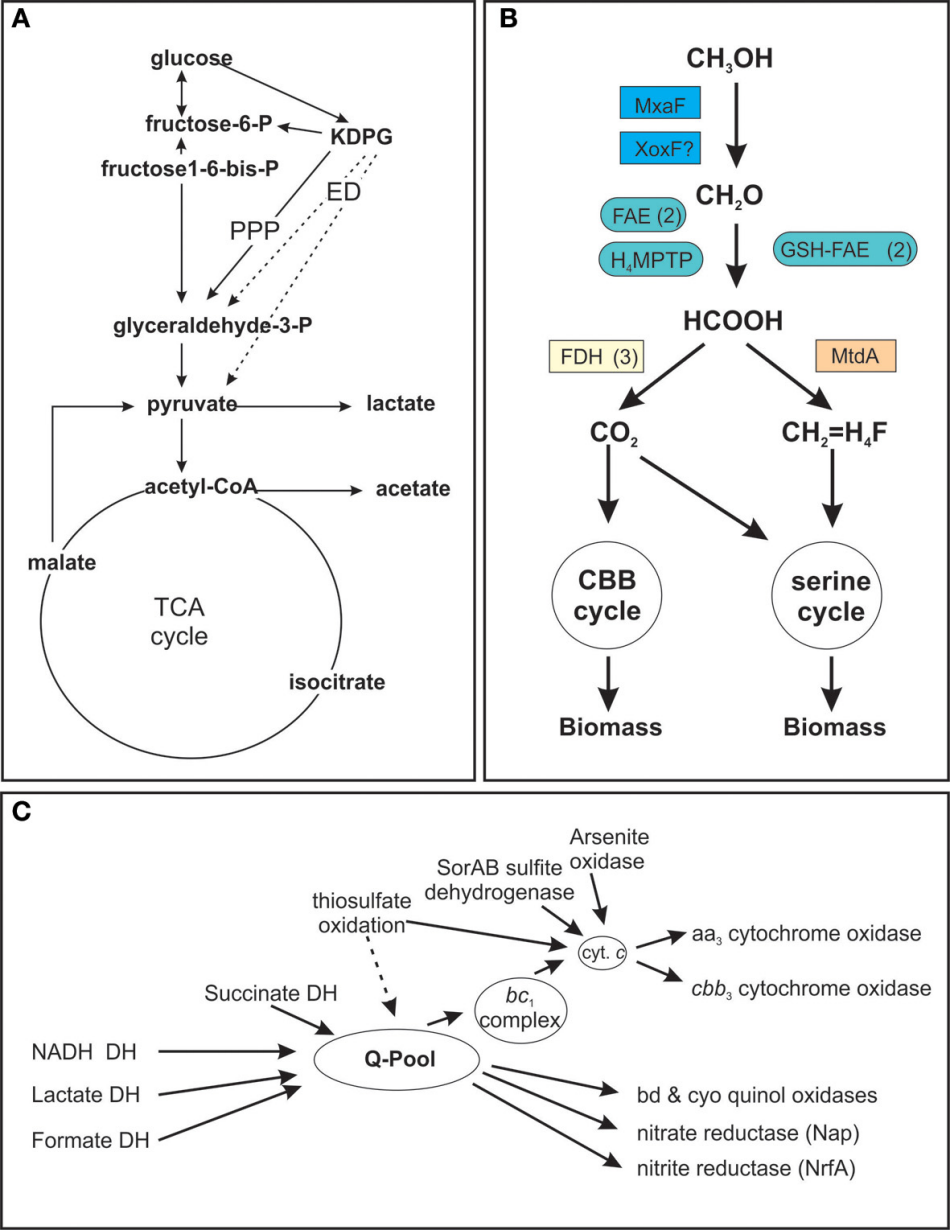
**Schematic representation of *S. novella* metabolic pathways. (A)** Glucose catabolism and TCA cycle **(B)** Methanol degradation **(C)** Respiratory chain components. **(A)** Dashed arrows denote reactions of the Entner Doudoroff (ED) pathway, PPP, Pentose Phosphate Pathway; DH, dehydrogenase, KDPG, 2-keto-3-deoxy-6-phosphocluconate. **(B)**: H4MPTP, methylene tetrahydromethanopterin pathway; MtdA, methylene tetrahydromethanopterin dehydrogenase; FAE, formaldehyde activating enzyme; GSH-FAE, Glutathione dependent formaldehyde activating enzyme; FDH, formate dehydrogenase. **(C)** Q-pool, quinone pool. Gene numbers for all components or the different pathways are listed in the supplementary tables. Carbon metabolism—Table S2; Respiratory chain—Table S3; methanol metabolism—Table S4.

The high degree of metabolic flexibility that is apparent in the large number of growth substrates that can be used by *S. novella* is also very clearly reflected in the pathways encoded in its genome (Kappler et al., 2012). A partial analysis of *S. novella* initial glucose catabolism and its respiratory chain was carried out as part of the original genome analysis and revealed the presence of a pentose phosphate pathway (PPP) as well as an Entner Doudoroff (ED) pathway for the degradation of glucose (Figure 1A) while the absence of phosphofructokinase (EC 2.7.1.146) in the glycolysis pathway indicates that its primary purpose is gluconeogenesis. In addition the presence of multiple oxygen-dependent terminal reductases in the *S. novella* respiratory chain was noted (Kappler et al., 2012). However, there were no previous analyses of any enzymes or pathways involved in pyruvate metabolism, methanol degradation, and only two gene clusters encoding sulfur oxidizing enzymes had been previously described.

### Pyruvate metabolism

The routes by which pyruvate produced by glucose breakdown can be utilized include oxidation to lactate via the action of one of several putative lactate dehydrogenases (Snov_0154, Snov_0198, Snov_1738, Snov_3299, Snov_4339) or to acetyl-CoA by a pyruvate dehydrogenase complex (genes Snov_1789 to Snov_1795).

Acetyl-CoA can then enter the TCA cycle, which in *S. novella* also includes a glyoxylate shunt, for complete oxidation to carbon dioxide or be converted to acetate via the action of phosphate acetyl transferase (Snov_4211) and acetate kinase (Snov_2209) (Figure 1A, Table S2).

### Composition of the respiratory chain

Following the complete oxidation of acetyl-CoA to carbon dioxide the resulting NADH/FADH_2_ can enter the respiratory chain (Table 1 and Table S2) which is composed of a complete complex I (NADH: ubiquinol oxidoreductase, EC 1.6.5.3, Snov_1849-Snov_1864), a succinate dehydrogenase (complex II, EC 1.3.5.1, Snov_3317-Snov_3320), and a cytochrome *bc*_*1*_ complex (complex III, EC 1.10.2.2, Snov_2477-Snov_2479) (Figure 1C, Table S2). In addition there are two more loci (Snov_2406-Snov_2408 and Snov_3849-Snov_3850) encoding three or two proteins, respectively, annotated as subunits of complex I, as well as two putative formate dehydrogenases (FDH) (Snov_3504-Snov_3507, put FDH-F type; Snov_3851-Snov_3852, put FDH-O/N type), an arsenite oxidase (Snov_1288-Snov_1289), and a putative LldD type lactate dehydrogenase (Snov_0680) that can also feed electrons into the respiratory chain.

**Table 1:**
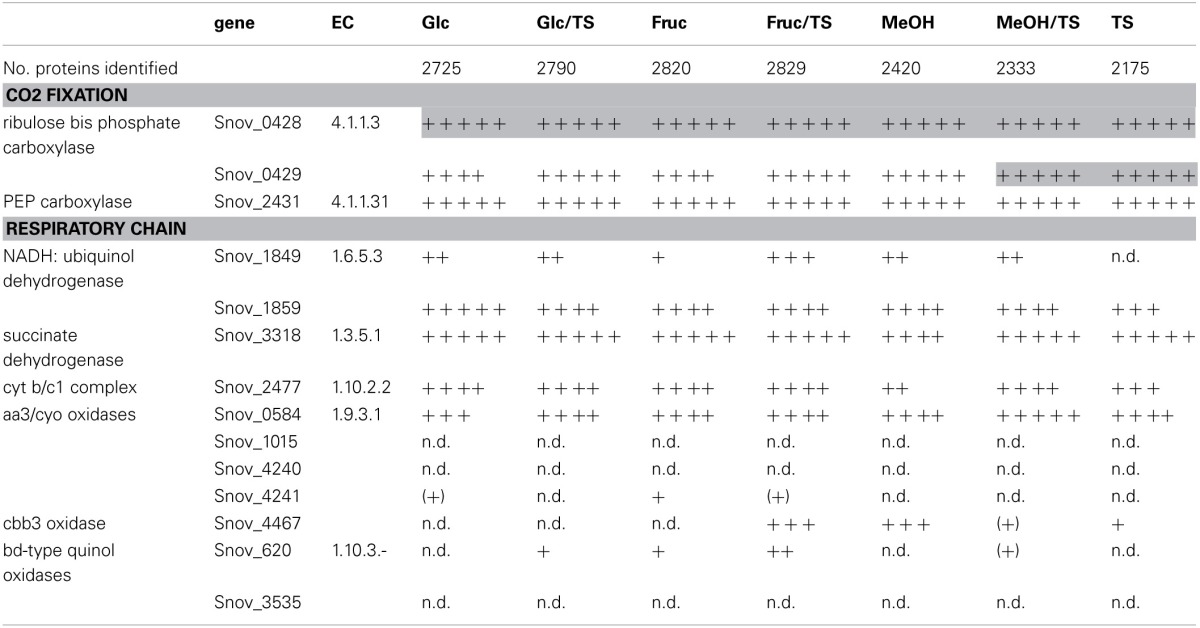
**Presence of proteins involved in carbon dioxide fixation and respiration in *S. novella* cell extracts from chemolithoautotrophic, methylotrophic, heterotrophic, and mixotrophic growth conditions**.

+, indicate expression levels, with the number of crosses indicating the band in which the protein was detected; +++++, top 10% of proteins; ++++, 10–30%; +++, 30–50%; ++, 50–70%; +, 70–90%; (+), 90–100%; n.d., not detected. Shading indicates that the protein was in the top 2% of proteins that were detected in the sample.

This large number of dehydrogenase enzymes that can feed electrons into the respiratory chain is linked to an array of terminal reductases that can mediate both aerobic and anaerobic respiration (Figure 1C). The final step in respiration can use at least six types of cytochrome or quinol oxidases, including two aa_3_ type oxidases (Snov_0584-Snov_0589 and Snov_4240-Snov_4243). The second of these two enzymes, Snov_4240 has similarity to both Cox1 (aa_3_ type oxidase) and CyoB oxidases with the similarity to the Cox1 enzymes being slightly higher. Despite this the current annotation classifies the enzyme as a CyoB-type oxidase. Another gene locus (Snov_1015-Snov_1019) also encodes proteins related to the heme Cu oxidases (cyoB-type quinol oxidases) and here one protein (Snov_1016) is a fusion of the subunit I and subunit III cytochrome oxidase domains. This type of cytochrome oxidase appears to be conserved in a variety of α-,β-, and γ-Proteobacteria as well as some Planktomycetes. A *cbb*_3_-type oxidase with high oxygen affinity (Snov_4464-Snov_4468) is present as well as two bd-type quinol oxidases (Snov_0619-Snov_0620 and Snov_3535-3536) which are also known for their high affinity to oxygen. Complementing the aerobic respiration are several terminal reductases known to be involved in anaerobic respiration such as nitrate reductase (Nap–type, Snov_1159-Snov_1162, EC 1.7.99.4), a cytochrome *c* dependent nitrite reductase (Snov_1147; EC 1.7.2.1), and a nitric oxide reductase (Snov_1155; EC 1.7.2.4) (Figure 1C). There are also several uncharacterized enzymes of the DMSO reductase enzyme family encoded in the *S. novella* genome (Kappler and Nouwens, 2013) which may also be linked to the respiratory chain.

### Metabolic pathways and genes involved in C1 metabolism in *S. novella*

While it has been known for over 30 years that *S.novella* can use methanol as a growth substrate, the relevant pathways and enzymes had never been studied. Our analyses showed that the ability of *S. novella* to oxidize methanol appears to be based on a combination of pathways and enzymes similar to those of a model methylotroph, *Methylobacterium extorquens* (reviewed in Chistoserdova, 2011). A full operon encoding an MxaF type methanol dehydrogenase (Snov_4185-Snov_4199, *mxaBxHFJGARSACKLDE*) was identified as well as a *xoxF* gene locus (Snov_1035-Snov_1038) encoding a putative methanol dehydrogenase-homolog (Figure 1B). While XoxF has been suggested to be involved in methanol oxidation in some bacteria, the exact function of this enzyme is still being investigated (Chistoserdova, 2011). Two copies each of genes encoding glutathione dependent (Snov_1125, Snov_1350) and independent putative formaldehyde-activating enzymes (FAEs) (Snov_0740, Snov_1050) (Vorholt et al., 2000; Goenrich et al., 2002) are present in the *S. novella* genome. These enzymes target the formaldehyde produced by the methanol dehydrogenases for further conversion via the tetrahydromethanopterin (TH_4_MP) pathway (Chistoserdova et al., 2009; Chistoserdova, 2011). For the assimilation of carbon units into cell biomass *S. novella* contains a complete serine pathway, as well as a tetrahydrofolate (TH_4_F) and a tetrahydromethanopterin (TH_4_MP) pathway (Tables 1, 3 and Table S2; Figure 1B). A complete Calvin Benson Bassham (CBB)-cycle for carbon dioxide fixation is also present (Table S2).

### Dissimilatory sulfur oxidation pathways

**Figure 2:**
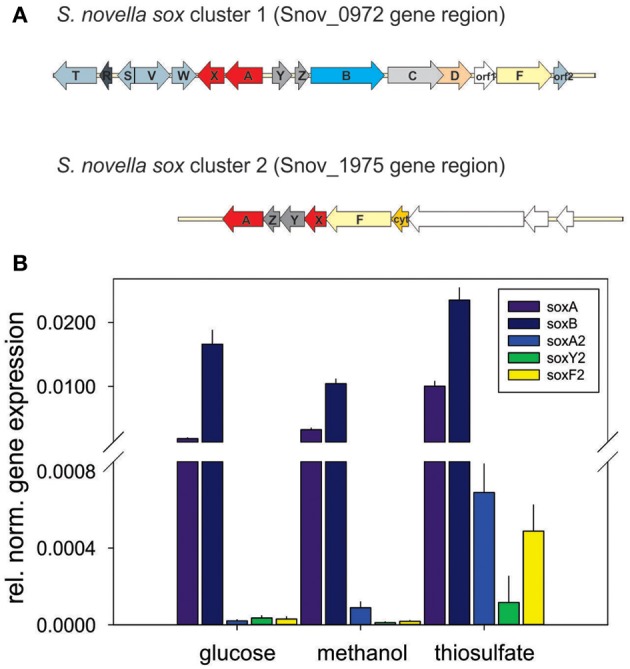
**Genes involved in dissimilatory sulfur oxidation in *S. novella*. (A)** Schematic representation of the two *sox* gene clusters encoded in the *S. novella* genome. *soxAX*, red; *soxYZ*, dark gray; *soxB*, light blue; *soxC*, light gray; *soxD*, orange; *soxF*, yellow; accessory genes (e.g., *soxV,W,S* etc.), blue gray; *soxR*, dark blue gray; *orf1* membrane anchor protein (main *sox* cluster) and Xanthine Oxidase family Mo enzyme (second *sox* cluster), no fill. **(B)** Expression of *sox* genes in *S. novella* cultures grown in the presence of glucose, methanol, or thiosulfate/CO_2_. Error bars represent the Standard Deviation of the mean, gene expression was normalized relative to the expression of the 16S RNA gene. A number “2” added to the gene name denotes that this gene is present in the second *sox* gene cluster.

Although the ability of *S. novella* to grow as a sulfur chemolithoautotroph was recognized at the time of its isolation (Starkey, 1935), details of the enzymes and pathways involved have only been elucidated recently. A gene region encoding the four core enzymes of a Sox-type sulfur oxidation pathway had been identified previously (Kappler et al., 2001, 2004) and analysis of the genome showed that this gene cluster (Snov_0978-Snov_0965) is more extensive than previously recognized. The full gene cluster contains 15 genes, *soxT(R)S-soxVW-soxAX-soxYZBCDorf1Forf2*, organized in at least four separate transcriptional units as indicated (Figure 2A).

Interestingly, in the current genome annotation the *soxR* gene that encodes a regulator of thiosulfate oxidation is marked as a pseudogene, possibly due to an N-terminal truncation of the encoded protein caused by a frameshift mutation at ~bp58-62 (sequence: CCCC) in the *soxR* gene that leads to a loss of 26 aa at the N-terminus of the protein (assuming translation of the truncated protein would start at the closest ATG codon). Analysis of the genome also revealed the presence of a second, smaller *sox* gene cluster (*soxX*_2_*Y*_2_*Z*_2_*A*_2_*F*_2_, genes Snov_1982-1977) which encodes duplicate copies of two of the core components of the Sox system (SoxAX, SoxYZ; the SoxB and SoxCD proteins are missing) (Figure 2A). This operon also contains genes encoding a molybdenum enzyme of the Xanthine Oxidase family (Snov_1975/Snov_1976) that was identified in an analysis of the molybdoproteome of *S. novella* (Kappler and Nouwens, 2013).

We also analyzed the presence of other enzymes capable of oxidizing a variety of reduced sulfur compounds. A Sox complex independent sulfite oxidizing enzyme, SorAB, had already been identified previously (Kappler et al., 2000) and the enzyme itself has been extensively characterized (Kappler and Bailey, 2005; Kappler et al., 2006, 2012; Rapson et al., 2008; Bailey et al., 2009; Emesh et al., 2009). No evidence was found for the presence of Sox complex independent sulfide oxidizing enzymes such as flavocytochrome *c* and sulfide:quinone reductase. Similarly, despite early observations of a GSH-dependent sulfur oxygenase activity in *S. novella* (Charles and Suzuki, 1966a), no homologs of bacterial sulfur oxygenases were identified using the *Acidithiobacillus caldus* enzyme (acc no ZP_0529337) as the search model.

### Adaptation of S. novella metabolism to the presence of various carbon and sulfur sources

In order to investigate the importance of the enzymes and pathways identified above for metabolic adaptation of *S. novella* we analyzed protein and gene expression in *S. novella* cultures grown under heterotrophic (glucose, fructose), methylotrophic (methanol), and sulfur chemolithoautotrophic (thiosulfate/carbon dioxide) conditions as well as under mixotrophic conditions where thiosulfate was combined with either a substrate for heterotrophic (glucose or fructose) or methylotrophic growth (methanol).

### Carbon metabolism

As might be expected, enzymes belonging to key pathways of central carbon metabolism were detected under all conditions tested using shotgun proteomics (Table S2). These included the pentose phosphate and glycolysis pathways (although the latter likely is used for gluconeogenesis rather than glucose oxidation), the pyruvate dehydrogenase complex and all enzymes of the TCA cycle (Table S2). Enzymes of the glyoxylate shunt were also always expressed but its key enzyme, isocitrate lyase (ICL), appeared to undergo some regulation in response to changing carbon sources, for example in the presence of glucose and during growth on thiosulfate ICL was not detected, and during growth on fructose ICL was in the bottom 5% of proteins detected (Table S2).

Phosphoenolpyruvate carboxylase, an enzyme catalyzing an anaplerotic reaction leading to CO_2_ fixation, was always present at high levels (within the top 10% of proteins detected) (Table 1), and in addition to the PPP which appeared to be the main pathway for the degradation of sugars, enzymes specific to the ED pathway were also detected, however, one of the key enzymes, 2-dehydro-3 deoxy-gluconate aldolase was not detected during growth on fructose, methanol or thiosulfate, suggesting that this pathway is not used during growth on these substrates (Table S2).

**Figure 3:**
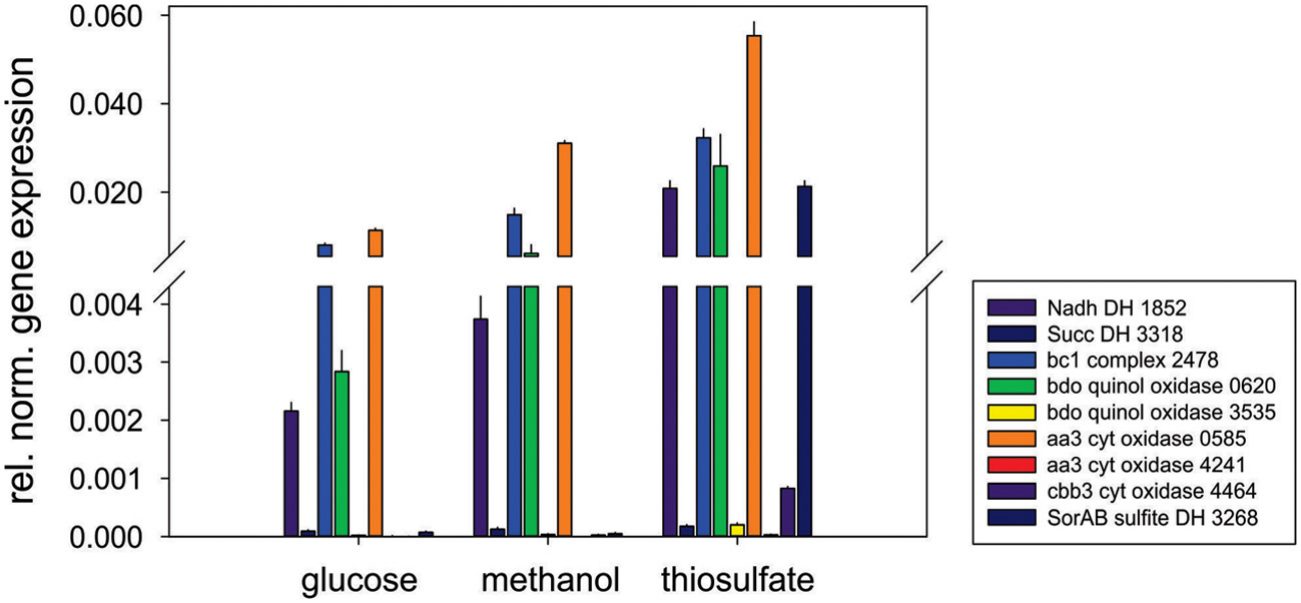
**Expression of respiratory chain genes in *S. novella* cultures grown under hetero-trophic, methylotrophic, or sulfur chemolithoautotrophic conditions**. Error bars represent the Standard Deviation of the mean, gene expression was normalized relative to the expression of the 16S RNA gene.

Respiratory chain complexes also showed very consistent patterns of expression with ATP synthase (Snov_4429-4433), complexes I (Snov_1852-Snov1864 gene region), II (succinate dehydrogenase, Snov_3317-Snov_3320) and the *bc*_*1*_ complex being present under all conditions tested (Table 1 and Table S3). Considerable variation in expression was, however, present for the six terminal oxidase complexes encoded in the *S. novella* genome. Of the aa_3_/cyo type oxidases only the aa_3_ –type enzyme encoded by the Snov_0584 gene region was detected at appreciable levels using shotgun proteomics. The enzymes encoded in the Snov_1015 and Snov_4241 gene regions were either completely absent or only present at low levels under specific conditions (Glc and Fruc, bottom 10% and 15% of proteins detected). The core subunits of the *cbb*_3_ terminal oxidase were only present when thiosulfate, methanol, methanol/thiosulfate or fructose/thiosulfate were used as growth substrates, while of the two *bd*-type quinol oxidases only the enzyme encoded by the Snov_0620 gene region was detected, mostly in samples from mixotrophic growth (Table 1). These results mostly match the qPCR data generated from cDNA of cultures grown on glucose, methanol or thiosulfate medium (Figure 3), with the exception of expression of the Snov_0620 encoded bd oxidase which showed comparatively high expression levels under the three conditions tested, while only low amounts of the corresponding proteins were detected. A possible explanation for the lower levels of detection for peptides could be the association of the respiratory chain complexes with the cell membrane.

### Dissimilatory sulfur metabolism

Monitoring of the expression of enzymes for chemolithotrophic growth using sulfur compounds revealed that the previously discovered *sox* operon (Snov_0978-Snov_0968) that encodes a complete Sox-type thiosulfate oxidation enzyme complex is the main operon involved in chemolithotrophic sulfur oxidation in *S.novella* (Table 2, Figure 2). All relevant proteins were detected in all proteome samples analyzed, and several core proteins of the enzyme complex (SoxB, SoxA, and Sox C) were always among the top 100 protein detected in the samples (Table 2). Levels of the flavocytochrome *c*-like SoxF protein were lowest relative to the other complex components (within the top 40–60% of proteins). We also detected peptides for a protein encoding a putative membrane anchor protein (DUF1791 type protein), Snov_0966 or Orf1, which had previously been suggested to act as a potential membrane anchor for the *S. novella* Sox complex (Kappler et al., 2004) which had been postulated by early biochemical studies (Aleem, 1965; Charles and Suzuki, 1966a; Kappler et al., 2004). Using qRT-PCR we also found evidence for the functionality of the *soxR* “pseudogene” for which expression was detected under all three conditions tested, with the highest levels detected in the presence of thiosulfate (Figure S1).

**Table 2:**
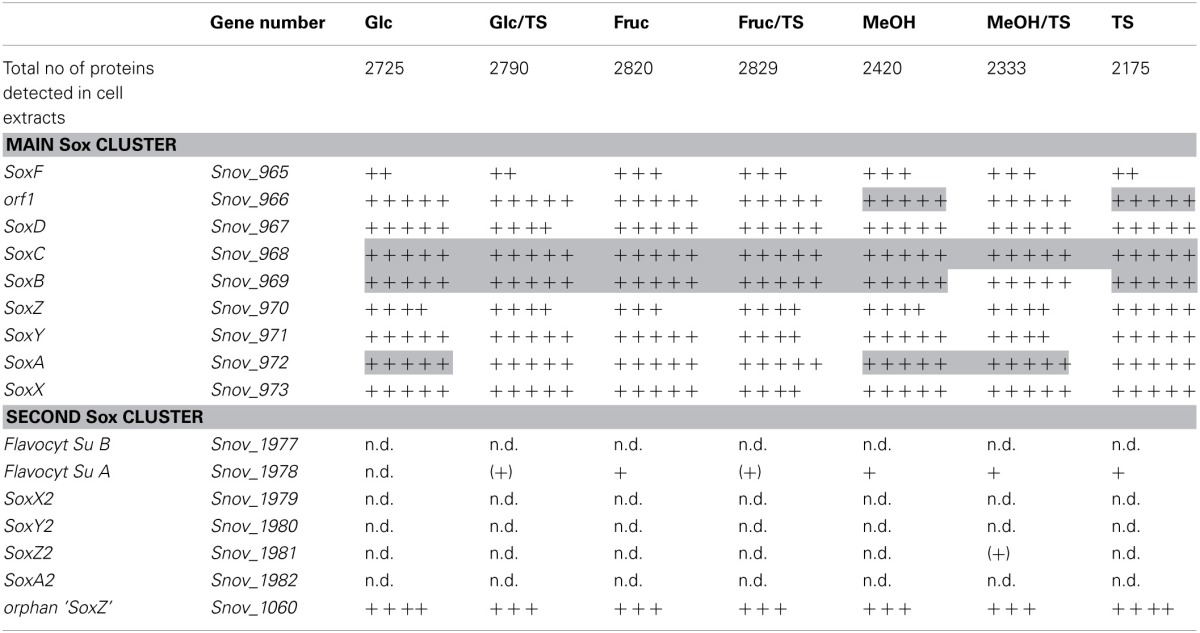
**Presence of proteins involved in sulfur chemolithotrophy in *S. novella* cell extracts from chemolithoautotrophic, methylotrophic, heterotrophic, and mixotrophic growth conditions**.

+, indicate expression levels, with the number of crosses indicating the band in which the protein was detected; +++++, top 10% of proteins; ++++, 10–30%; +++, 30–50%; ++, 50–70%; +, 70–90%; (+), 90–100%; n.d., not detected. Shading indicates that the protein was in the top 2% of proteins that were detected in the sample.

In contrast the SoxAX and SoxYZ proteins encoded by the second *sox* gene cluster (Snov_1982-Snov_1979) were not detected, although one subunit of the SoxF-like flavocytochrome *c* protein (Snov_1977-Snov_1978) was present at low levels (~bottom 20% of protein detected) throughout except following growth on glucose. A SoxZ-like protein encoded by a single gene (Snov_1060) was detected among the top 25–50% of proteins throughout, but the function of this protein is unclear as it is encoded by a gene that does not appear to form an operon with any of the adjacent genes, and none of these encode a SoxY*-*like protein.

These results are in agreement with qRT-PCR experiments that also showed high expression for genes encoded in the main *sox* cluster, and very low expression levels for proteins from the second *sox* gene cluster (Figure 2B). Both gene expression and proteomic data clearly showed that the SorAB sulfite dehydrogenase (Snov_3268/3269) is induced by the presence of thiosulfate in the growth medium regardless of the carbon source present as already reported in Kappler and Nouwens (2013).

### Metabolism of C1-compounds

*S. novella* has been classified as a facultative methylotroph since the discovery of its ability to oxidize methanol, however, similar to what was observed for sulfur chemolithotrophic growth, the MxaF methanol dehydrogenase was one of the five most abundant proteins detected under all conditions tested except when fructose was included in the medium where MxaF was among the top 20 proteins detected. This clearly indicates that together with sulfur chemolithotrophy, methylotrophy is another key growth mode for *S. novella* and it is also similar to what was found in *M. extorquens* (Bosch et al., 2008). While all proteins of the *mxa* operon were detected in all samples, a slight increase in protein abundance was observed when methanol was the growth substrate (Table 3 and Table S4). The second possible methanol dehydrogenase, the XoxFGJ protein, was also detected in all samples. XoxF was usually found in the top 100–200 proteins detected, except when methanol or thiosulfate were the growth substrates when XoxF was ranked approx. 70 out of over 2000 detected proteins. This indicates a role for this protein in methylotrophic and possibly also sulfur chemolithotrophic growth.

**Table 3:**
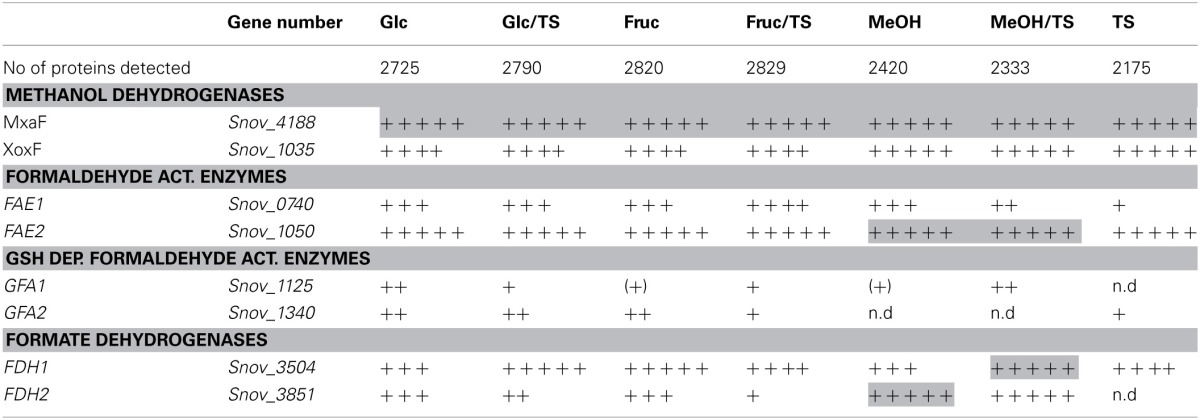
**Presence of proteins involved in methylotrophy *S. novella* in cell extracts from chemolithoautotrophic, methylotrophic, heterotrophic and mixotrophic growth conditions**.

+, indicate expression levels, with the number of crosses indicating the band in which the protein was detected; +++++, top 10% of proteins; ++++, 10–30%; +++, 30–50%; ++, 50–70%; +, 70–90%; (+), 90–100%; n.d., not detected. Shading indicates that the protein was in the top 2% of proteins that were detected in the sample.

**Figure 4:**
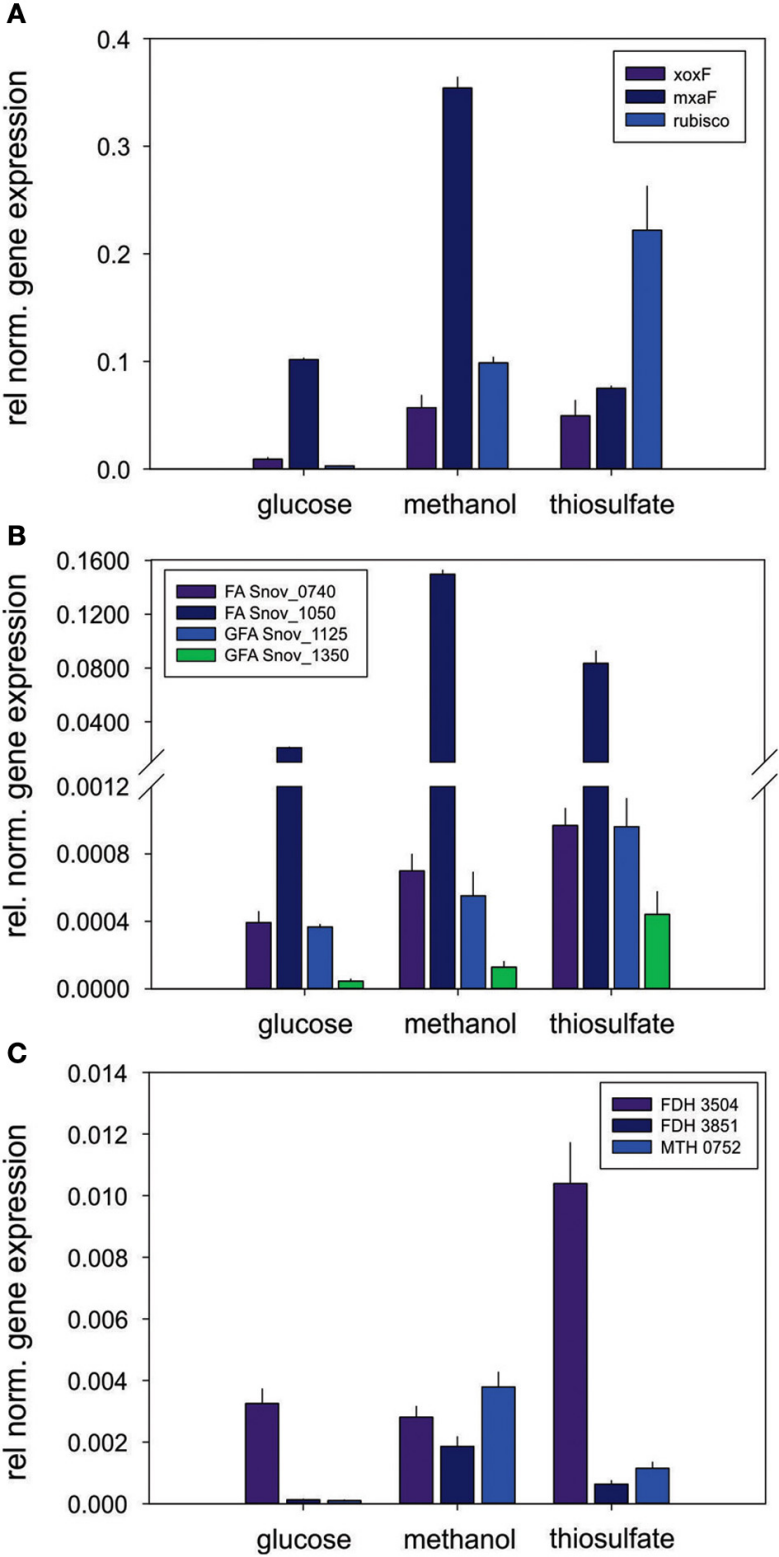
**Expression of genes involved in methylotrophy in *S. novella*. (A)** Methanol oxidation and carbon dioxide fixation (Calvin cycle), **(B)** Formaldehyde activating enzymes, **(C)** Formate dehydrogenases. Error bars represent the Standard Deviation of the mean, gene expression was normalized relative to the expression of the 16S RNA gene.

Other enzymes involved in methylotrophy such as the two glutathione dependent FAEs were either expressed at low levels (~bottom 30% -bottom 5% of proteins detected) or not at all, while of the two GSH - independent FAEs the Snov_0740 enzyme was always among the top 40–50% of protein detected and showed no obvious substrate dependent regulation. In contrast, the Snov_1050 encoded FAE was among the top 10% of proteins detected, and in the presence of methanol and/or thiosulfate the relative abundance of the protein increased (top 3% of proteins detected) clearly linking this enzyme to methylo- and chemolithotrophic growth (Table 3 and Table S4). The qRT-PCR data also showed an increase in Snov_1050 transcripts in the presence of methanol and thiosulfate (Figure 4).

A similar observation was made for the FDHs where the Snov_3851 encoded FDH was clearly induced in the presence of methanol (protein abundance increased from the top 40–50% to the top 5% of proteins), and the Snov_3504 FDH showed increased abundance in thiosulfate and methanol/thiosulfate containing samples. Again, the overall patterns observed for the gene expression data matched the shotgun proteomic data (Figure 4, Table 3). For further assimilation of the C1 units the serine pathway, the methylene tetrahydromethanopterin pathway and the enzymes of the Calvin cycles were expressed in *S. novella* under all conditions (Figure 3, Table 1 and Table S2), with the abundance of enzymes of the latter pathway increasing in the presence of methanol, thiosulfate or a combination of the two.

## Discussion

Although several proteomic studies of soil bacteria, their interactions with plants and their responses to a variety of environmental stresses (e.g., heavy metals) have been carried out (Hansmeier et al., 2006; Cheng et al., 2010; Ray et al., 2013; Van Dijl and Hecker, 2013), the interplay between different growth modes associated with carbon and sulfur metabolism have not previously been investigated in a free living soil bacterium despite their significance for global element cycles and the speciation of carbon and sulfur compounds in the environment.

The data presented above clearly demonstrate that in *S. novella*, the pathways associated with sulfur chemolitho- and methylotrophic growth modes are constantly expressed at high levels regardless of the carbon sources present and independent of auto-, mixo- or heterotrophic growth conditions. Some form of substrate dependent regulation appeared to occur for some pathway components, but in general key enzymes were found to be highly abundant. This then indicates that both methylotrophy and sulfur chemolithotrophy are key growth modes for *S. novella* which in turn raises the question whether *S. novella* should be classified as a facultative methylo- and chemolithotroph. It also appears that in the presence of methanol and thiosulfate *S. novella* does employ a mixotrophic growth strategy as cultures grown in the presence of both substrates reached culture densities that were at least twice as high as the maximum values obtained after growth on thiosulfate, indicating that methanol must have been utilized by the bacteria to increase cell growth, and cultures grown on methanol and thiosulfate showed a strong thiosulfate dependent respiratory activity in experiments using an oxygen electrode (Kappler, unpublished).

Enzymes for various pathways allowing CO_2_ fixation were also expressed under all growth conditions tested, and this included the anaplerotic reaction mediated by PEP carboxylase as well as enzymes of the Calvin and serine cycle, which suggests a role for CO_2_ fixation processes in balancing metabolic fluxes and possibly also redox states. Especially the enzymes of the Calvin cycle were highly abundant in *S. novella* under all conditions tested, indicating that it might be the major pathway for carbon dioxide fixation.

Our results are in contrast to a study by Lejohn et al. (1967) who reported catabolite repression of thiosulfate oxidation in the presence of glucose, lactate, glycerol, ribose, and pyruvate, while several amino acids, including glutamate were reported not to cause any inhibition. The results of our work agree, however, with data of a later study (Perez and Matin, 1980) which found that glucose and thiosulfate were oxidized concurrently to carbon dioxide and sulfate by *S. novella* independent of the relative concentrations of glucose and thiosulfate. As both of these studies as well as our work used the same strain of *S. novella* (DSMZ506^T^ = ATCC 8093^T^) and very similar mineral media for the cultivation of the bacteria it is unclear what caused the observed difference in substrate utilization. Another study that investigated *S. novella* substrate utilization under nutrient limiting conditions in continuous culture (Leefeldt and Matin, 1980) also found a concurrent utilization of substrates.

In this work, proteins involved in central carbon metabolism pathways of *S. novella* were detected under all growth conditions tested, and our data thus agree with enzymatic studies of *S. novella* physiology that indicated that a complete TCA and glyoxylate cycle were present under both auto- and heterotrophic growth conditions (Charles, 1971). The presence of PEP carboxylase and RubisCO under all growth conditions tested also agrees with data from an earlier study (McCarthy and Charles, 1974), although neither the proteomic nor the qRT-PCR data detected the regulatory pattern observed by McCarthy and Charles (1974). McCarthy and Charles (1974) reported that autotrophically grown cells had much higher levels of RubisCO activity and lower levels of PEP carboxylase activity relative to heterotrophically grown cells while our data clearly indicate nearly invariant, high expression levels for both enzymes in the top 10% of proteins detected in each sample. A possible explanation for this difference could be that e.g., the enzymatic activity of RubisCO can be modulated by protein modifications and intracellular inhibitors (Jouanneau and Tabita, 1987; Wang and Tabita, 1992; Tabita, 1999), and thus the amount of protein present in the cell would not necessarily reflect the level of enzyme activity observed.

Another interesting observation is that while there are two gene clusters encoding components of a Sox-type thiosulfate oxidation pathway, only the gene cluster encoding the complete Sox complex was expressed at significant levels. This suggests that in *S.novella* the additional components of the Sox multienzyme system encoded in the second *sox* gene cluster have no functional significance as had been suggested e.g., for modulating substrate specificities through the use of isoenzymes of the SoxAX cytochromes (Frigaard and Dahl, 2008; Gregersen et al., 2011; Kappler and Maher, 2013). As at present not much is known about the roles of duplicate *sox* genes for microbial physiology the results presented here should be followed up by additional work on other bacteria that contain gene loci encoding multiple copies of *sox* genes to confirm the lack of a functional role. It is also possible that the second *sox* gene cluster in *S. novella* is important under growth conditions other than those tested here.

Of additional interest is the putative SoxR regulator encoded in the main *sox* gene cluster. Although classified in the genome annotation as a pseudogene, qRT-PCR (Figure S1) clearly indicated that the gene is functional and would presumably give rise to a functional SoxR protein. Given that regulation of expression of the main *S. novella sox* gene cluster happens at a very high level of expression it will be interesting to determine possible functional differences between the truncated SoxR regulator from *S. novella* and the characterized, full length SoxR regulators from *P. pantotrophus* and *Pseudoaminobacter salicylatoxydans* (Rother et al., 2005; Mandal et al., 2007).

Redundancy of genes encoding various elements of the degradation pathway also characterizes the methylotrophy pathways in *S. novella* (Table 3 and Table S4, Figure 1), and based on our data it was possible to clearly assign a role in methylotrophy to some of the redundant systems, such as the MxaF methanol dehydrogenase which appears to be the main methanol oxidizing enzyme, the Snov_1050 encoded FAE and the Snov_3851 encoded FDHs which were clearly methanol inducible.

Overall our results indicate that *S. novella* employs a mixotrophic growth strategy, in which several pathways for “specialized” types of metabolism such as the utilization of C1-compounds and dissimilatory energy generation from sulfur compounds are always expressed at high levels. This is in contrast to studies on other bacteria such as *Paracoccus pantotrophus* where thiosulfate oxidation was induced by the presence of thiosulfate (Robertson and Kuenen, 1983; Chandra and Friedrich, 1986; Rother et al., 2005) but is reminiscent of what has been reported for *M. extorquens*, where methanol and succinate were found to be co-metabolized and enzymes involved in methylotrophy were always expressed at high levels (Bosch et al., 2008; Peyraud et al., 2012).

It would then appear that the classification of *S. novella* as a facultative sulfur chemolithoauto- and methylotroph (Starkey, 1935; Kelly et al., 2000) does not accurately reflect the growth strategy employed by this bacterium. At this stage we can only speculate on the possible advantages inherent in this, but it is obvious that in such a bacterium production or sequestration/degradation of greenhouse active substances such as methanol or carbon dioxide would depend on the exact growth conditions encountered and the net contribution of *S. novella* to these processes might change very quickly in response to a changing environment, and soils are known to undergo significant fluctuations in many environmental parameters including oxygen and nutrient availability. This has direct implications for the modeling of microbially mediated climate relevant processes in soils, and also raises the question whether bacteria related to *S. novella* might use a similar combination of growth modes.

*S. novella* is a member of the family *Xanthobacteraceae* and within this family it is most closely related to *Ancylobacter* sp. which have been isolated from soils and waterbodies and are known methylotrophs (Kelly et al., 2000; Xin et al., 2006). No complete genome sequences for *Ancylobacter* sp. are available at present, and the description of most known *Ancylobacter* sp. does not mention whether their ability to oxidize thiosulfate was tested and this trait is not mentioned in the original species description or Bergey's Manual of Systematic Bacteriology (Larkin et al., 1977; Raj, 1989; Garrity et al., 2005; Xin et al., 2006). However, in 1998 several isolates of thiosulfate oxidizing soil bacteria were identified as *Ancylobacter* sp. (Stubner et al., 1998) and a recent description of a new *Ancylobacter* species, *A. dichloromethanicus* mentions growth as a facultative thiosulfate oxidizer as a trait of the species (Firsova et al., 2009). Similarly, *Xanthobacter* species including *X. autotrophicus* are known to be able to derive energy from thiosulfate oxidation, but the trait is not part of the species description, while the utilization of methanol as a growth substrate is recognized as a trait of the species (Padden et al., 1998; Stubner et al., 1998; Garrity et al., 2005).

It would thus appear that the organisms of the family *Xanthobacteraceae* not only share similar habitats (soils, plant root systems, and freshwater, including sediments) but also share many key metabolic traits, and it will be interesting to investigate whether representatives of other species within the family share any of the regulatory features uncovered here for *Starkeya novella*. Data of this type will be of prime importance for understanding mineralization processes in soil environment as well as the impact of these processes on climate relevant processes.

### Conflict of interest statement

The authors declare that the research was conducted in the absence of any commercial or financial relationships that could be construed as a potential conflict of interest.

## Supplementary material

Figure S1 **Expression of the *S. novella* soxR “pseudogene” in cultures grown with glucose, methanol of thiosulfate as energy sources**. Error bars represent the standard deviation of the mean. Expression data were normalized relative to the expression of the 16S gene.
