# Supplementary figures and images for: Metabolic adaptation and trophic strategies of soil bacteria—C1- metabolism and sulfur chemolithotrophy in Starkeya novella
Source: Front Microbiol. 2013 Oct 17;4:304. doi: 10.3389/fmicb.2013.00304 (PMC3797975; doi:10.3389/fmicb.2013.00304)

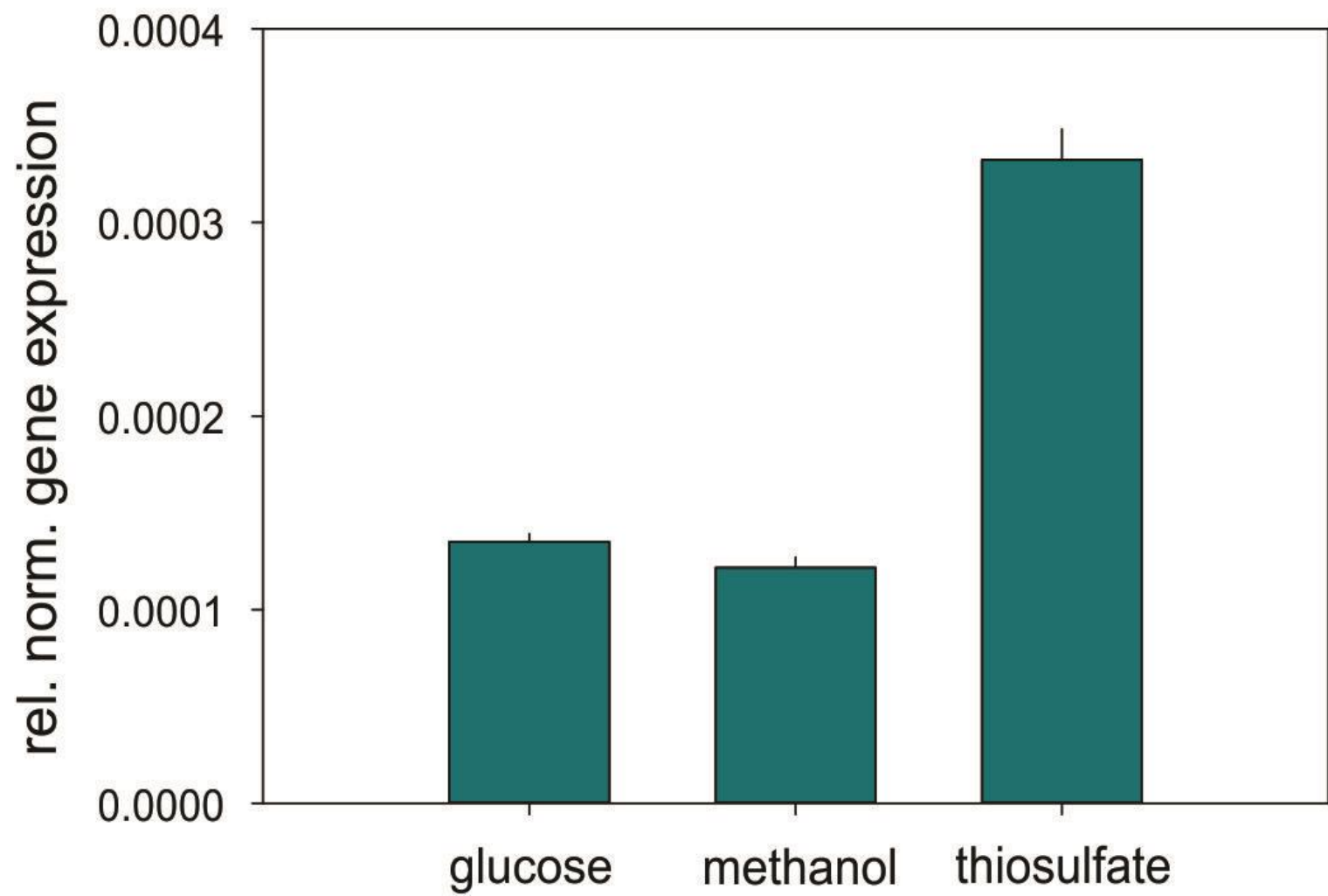

Supplement: Figure S1 — Expression of the S. novella soxR “pseudogene” in cultures grown with glucose, methanol of thiosulfate as energy sources. Error bars represent the standard deviation of the mean. Expression data were normalized relative to the expression of the 16S gene. [file Presentation_1.PDF]
