# Supplementary material for: Metabolic adaptation and trophic strategies of soil bacteria—C1- metabolism and sulfur chemolithotrophy in Starkeya novella
Source: Front Microbiol. 2013 Oct 17;4:304. doi: 10.3389/fmicb.2013.00304 (PMC3797975; doi:10.3389/fmicb.2013.00304)
Supplement: Supplementary file 2 [file DataSheet1.PDF]

gene name/number	forward primer	reverse primer
xoxF	GAACATCGTCTATGCGCTCGACCTG	GGCAAGGCCACGATTGACCG
mxaF	ACACCGACAACTGGCCGATGAC	ACGCCGGTGGAGAACGACCA
Snov_3504	CACCACGGAAACGCGAAACACC	CGCATCGCCCTCGATATGGA
Snov_3851	GTCATGCCGAAGGACGAGTCCAA	CGGAAATCGTCAGCGCGAAC
Snov_1125	CGGCGTGAAGGCGAGGATCA	ATTCACCGGCGGCTGCCATT
Snov_1050	GCCTGCTTGGTGGCCTCGTAGTTG	AAGGCGTGATCCCCGAGGAA
Snov_1350	GGCGACGAGGTCGAGGTGAATC	AATGGTGCGCGAGCGGGAAC
Snov_0740	ATATCTGGTTCCGCACCGGCG	ATCGAAGCGAAGGCGTGCCC
Snov_0752	AGTGATCGCGCCAGGAGGAAGG	GCGCACTGACCATTGGCCAT
Snov_0429	CTCGATCTCCAACCTGTCGGCC	CGGGCCGTCGAAGGTCTTCA
Snov_0585	GATCCGACGGGTTGGAAGCG	TGCGAATGCCGATGGAGAGG
Snov_0620	GGTGACGAAACCGATCGGCC	CGGCCTGCTGATGATCGCGA
Snov_1852	GGTGACGAAACCGATCGGCC	CGGCCTGCTGATGATCGCGA
Snov_2478	GATGCACACCGCCACCCAGA	CATGTTCGGCGCGATCATCG
Snov_3318	TTCTCGCCGGAGGTGAGCAGCT	AATGCGCCTGCGTCTCCGTG
Snov_3535	TTCTCGCCGGAGGTGAGCAGCT	AATGCGCCTGCGTCTCCGTG
Snov_4239	GATCAGATCAGGCAGACCTTCGGC	CCTTGAGATCGGCGACGGTG
Snov_4464	GGGTCGCATGAGGCAAGAGATCG	TATGGCGAAGACGACGGCGA
soxA2	AGCTCGATCTCGCCTGCTCGTCCT	ACCGCCTGCCATTCCAGCCGATA
soxX2	CCGCACCTGCAGGGCAATCT	TCGGCATCAGGCTGTCGGGA
soxF2	ACTACACCGCCTGCCCGATGA	ACCACACGCTGCGGCACATG
soxB	TGACGGAAGGCTCACGGAATAGA	ACCGGCAAGATGACGCAGGA
soxF	TGCCGTCCTCCAGCTTGATGGT	GCGGCGGATTCGATCTGGTT
soxA	ATC TCG TCG CCT TCA TCG CCA	ATC GAG GAG CGC CGG AAG AA
sorA	AGC ATT GAT TGA CGG CGA CGA	ATC GAC CCC GAT GCA TTC CG
soxY	GTG CAC CGC CTT CAC GTA ATC C	GTA GGC GGA CGG CAA GAT CA
16S 100 bp	TGG CAG ACG GGT GAG TAA CAC GT	GCG GGT TCA TCC AAT GGC GA
soxR	AGCTCGAGAACATCCTGTCGCTGC	TCGGCGAGGCTGTAGTAGACGG
16S 200 bp	AGGCGGATTGTTAAGTCAGGGGTG	TTTGCTCCCCACGCTTTCGC
