# Supplementary material for: Metabolic adaptation and trophic strategies of soil bacteria—C1- metabolism and sulfur chemolithotrophy in Starkeya novella
Source: Front Microbiol. 2013 Oct 17;4:304. doi: 10.3389/fmicb.2013.00304 (PMC3797975; doi:10.3389/fmicb.2013.00304)
Supplement: Supplementary file 3 [file DataSheet2.PDF]

	gene locus	EC number	Glc	Glc/TS	Fruc	Fruc/TS	MeOH	MeOH/TS	TS
total proteins detected in the sample			2725	2790	2820	2829	2420	2333	2175
data are reported in percent as the relative position of the proteins in the list of proteins detected, with 1.0 or 100% indicating the lowest ranking									
**glycolysis**									
hexokinase	Snov_2818	2.7.1.2	0.546	0.328	0.370	0.456	0.746	0.614	0.417
glucose-6-P isomerase	Snov_2251	5.3.1.9	0.121	0.063	0.061	0.056	0.031	0.054	0.053
fructose-1,6, bisphosphatase (glconeogenesis)	Snov_1366	3.1.3.11	0.057	0.096	0.091	0.079	0.323	0.168	0.100
phosphofructokinase	not annotated								
Fructose_bisphosphate aldolase	Snov-0427	4.1.2.13	0.063	0.054	0.095	0.076	0.036	0.013	0.011
	Snov_3072		0.032	0.018	0.023	0.019	0.017	0.015	0.011
triosephosphate isomerase	Snov_1812	5.3.1.1	0.567	0.479	0.805	0.522	0.484	0.359	0.382
glyceraldehyde -3-P dehydrogenase	Snov_1293	1.2.1.12	0.817	0.696	0.646	0.749			
	Snov_3070		0.018	0.009	0.037	0.016	0.005	0.003	0.004
phosphoglycerokinase	Snov_3071	2.7.2.3	0.052	0.032	0.072	0.027	0.012	0.009	0.009
phosphoglyceromutase	Snov_0324	5.4.2.1	0.099	0.070	0.082	0.072	0.067	0.070	0.062
Enolase	Snov_1800	4.2.1.11	0.029	0.057	0.029	0.028	0.028	0.026	0.029
pyruvate kinase	Snov_2767	2.7.1.40	0.635	0.916	0.857	0.308	0.632	0.711	0.807
	Snov_3164		0.065	0.072	0.113	0.131	0.228	0.145	0.184
PEP synthase (Same rxn as pyr kinase)	Snov_4007	2.7.9.2	0.009	0.005	0.006	0.004	0.015	0.007	0.015
**Pentose phosphate cycle**									
glucose-6-P -1- dehydrogenase (zwf)	Snov_3398	1.1.1.49	0.131	0.147	0.070	0.068	0.143	0.096	0.230
	Snov_3856		0.127	0.311	0.184	0.174	0.189	0.346	0.343
6-phosphoglucono lactonase	Snov_3399	3.1.1.31	0.334	0.436	0.285	0.387	0.455	0.303	0.304
6-phosphogluconate dehydrogenase	Snov_0228	1.1.1.44	0.705	0.720	0.762	0.650	0.874	0.608	0.523
	Snov_2639								
	Snov_3857		0.168	0.161	0.144	0.093	0.051	0.132	0.311
ribose-5 phosphate isomerase	Snov_0900	5.3.1.6	0.346	0.494	0.414	0.381	0.285	0.495	0.253
Ribulse phosphate 3 epimerase	Snov_0431	5.1.3.1	0.611	0.270	0.383	0.291	0.407	0.190	0.491
	Snov_2141								
transketolase	Snov_0426	2.2.1.1	0.054	0.014	0.067	0.025	0.014	0.005	0.006
	Snov_3069		0.025	0.022	0.016	0.036	0.036	0.041	0.033
	Snov_1793		0.049	0.115	0.059	0.046	0.025	0.022	0.307
	Snov_3205		0.047	0.148	0.024	0.068	0.024	0.135	0.458
transaldolase	Snov_3987	2.2.1.2	0.194	n.d.	0.187	0.148	0.206	0.141	0.272
**Entner Doudoroff Pathway I**									
glucose-6-P -1- dehydrogenase (zwf)	Snov_3398	1.1.1.49	0.131	0.147	0.070	0.068	0.143	0.096	0.230
	Snov_3856		0.127	0.311	0.184	0.174	0.189	0.346	0.343
6-phosphoglucono lactonase	Snov_3399	3.1.1.31	0.334	0.436	0.285	0.387	0.455	0.303	0.304
6-phosphogluconate dehydratase	Snov_3400	4.2.1.12	0.273	0.128	0.217	0.144	0.178	0.283	0.177
2-dehydro-3-deoxygluconate aldolase	Snov_2999	4.1.2.14	0.739	0.611	n.d.	0.714	n.d.	0.684	n.d.
**Pyruvate metabolism**									
**(A) to acetyl-CoA**									
Snov_1791 dihydrolipoamide dehydrogenase	Snov_1791		0.067	0.081	0.061	0.052	0.082	0.115	0.192
Snov_1792 pyruvate dehydrogenase complex dihydrolipoamide acetyltransferase	Snov_1792		0.030	0.077	0.082	0.034	0.058	0.084	0.241
Snov_1793 Transketolase central region [Starkeya novella DSM 506]	Snov_1793		0.049	0.115	0.059	0.046	0.025	0.022	0.307
Snov_1795 pyruvate dehydrogenase (acetyl-transferring) E1 component,	Snov_1795		0.140	0.091	0.065	0.032	0.081	0.090	0.365
**TCA cycle**									
citrate synthase	Snov_1825	2.3.3.1	0.041	0.049	0.094	0.042	0.059	0.069	0.061
aconitate hydratase	Snov_3489	4.2.1.3	0.005	0.004	0.003	0.005	0.011	0.009	0.006
isocitrate DH	Snov_2160	1.1.1.42	0.006	0.008	0.008	0.008	0.006	0.012	0.009
2-oxoglutarate DH	Snov_3300		0.007	0.008	0.004	0.006	0.010	0.016	0.007
Succ.-CoA synthase	Snov_2989	6.2.1.5	0.217	0.139	0.138	0.113	0.290	0.223	0.423
betaSU	Snov_2988		0.012	0.028	0.026	0.018	0.024	0.045	0.034
Succinate Dh Falvoprotein SU	Snov_3318	1.3.5.1	0.066	0.052	0.039	0.041	0.102	0.091	0.095
Fe/S subunit	Snov_3317		0.135	0.322	0.246	0.221	0.312	0.349	0.467
fumarate hydratase	Snov_3100	4.2.1.2	0.414	0.267	0.230	0.145	0.229	0.476	0.416
Malate/lactate dehydrogenase	Snov_4339	1.1.1.37	n.d.	0.817	0.930	n.d.	0.754	1.089	n.d.
	Snov_3299		0.025	0.117	0.087	0.117	0.053	0.117	0.069
	Snov_1738		n.d.	n.d.	n.d.	n.d.	n.d.	n.d.	n.d.
	Snov_0198		1.014	0.834	0.740	0.932	1.000	0.891	0.762
	Snov_0154		0.563	0.627	0.427	0.673	0.386	0.587	0.341
**glyoxylate shunt**									
isocitrate lyase	Snov_0815	4.1.3.1	n.d.	0.813	1.024	0.054	0.469	0.997	n.d.
malate synthase	Snov_0814	2.3.3.9	0.148	0.577	0.051	0.055	0.300	0.710	0.741
PEP carboxylase	Snov_2431	4.1.1.31	0.092	0.040	0.083	0.070	0.065	0.087	0.074
