# Supplementary material for: Metabolic adaptation and trophic strategies of soil bacteria—C1- metabolism and sulfur chemolithotrophy in Starkeya novella
Source: Front Microbiol. 2013 Oct 17;4:304. doi: 10.3389/fmicb.2013.00304 (PMC3797975; doi:10.3389/fmicb.2013.00304)
Supplement: Supplementary file 4 [file DataSheet3.PDF]

	gene locus	EC number	Glc	Glc/TS	Fruc	Fruc/TS	MeOH	MeOH/TS	TS
total proteins detected in the sample			2725	2790	2820	2829	2420	2333	2175
data are reported in percent as the relative position of the proteins in the list of proteins detected, with 1.0 or 100% indicating the lowest ranking									
**Calvin cycle**									
ribulose bis phosphate carboxylase	Snov_0428	4.1.1.3	0.017	0.003	0.010	0.007	0.003	0.002	0.000
	Snov_0429		0.250	0.045	0.243	0.069	0.045	0.019	0.024
phosphoglycerate kinase	Snov_3071	2.7.2.3	0.052	0.032	0.072	0.027	0.012	0.009	0.009
glyceraldehyde 3 phosphate DH	Snov_3070	1.2.1.13	0.018	0.009	0.037	0.016	0.005	0.003	0.004
triose_P_isomerase	Snov_1812	5.3.1.1	0.567	0.479	0.805	0.522	0.484	0.359	0.382
seduheptulose bisphosphate aldolase	Snov_0427	4.1.2.-	0.063	0.054	0.095	0.076	0.036	0.013	0.011
fructose bisphosphate aldolase	Snov_0427	4.1.2.13	0.063	0.054	0.095	0.076	0.036	0.013	0.011
	Snov_3072		0.032	0.018	0.023	0.019	0.017	0.015	0.011
inositolP/ fructose 1,6 bis phosphatase	Snov_0424	3.1.3.11	0.318	0.084	0.263	0.081	0.037	0.015	0.044
	Snov_1366		0.057	0.096	0.091	0.079	0.323	0.168	0.100
seduheptulosebis phosphatase	Snov_1366	3.1.3.37	0.057	0.096	0.091	0.079	0.323	0.168	0.100
transketolase	Snov_0426	2.2.1.1	0.054	0.014	0.067	0.025	0.014	0.005	0.006
	Snov_1793		0.049	0.115	0.059	0.046	0.025	0.022	0.307
	Snov_3069		0.025	0.022	0.016	0.036	0.036	0.041	0.033
	Snov_3205		0.047	0.148	0.024	0.068	0.024	0.135	0.458
ribulose phosphate 3 epimerase	Snov_0431	5.1.3.1	0.611	0.270	0.383	0.291	0.407	0.190	0.491
	Snov_2141		n.d.	n.d.	n.d.	n.d.	n.d.	n.d.	n.d.
ribose-5-P isomerase	Snov_0900	5.3.1.6	0.346	0.494	0.414	0.381	0.285	0.495	0.253
**CO2 fixation into oxaloactetate**									
carbonic anhydrase	Snov_1031	4.2.1.1	0.240	0.462	0.192	0.282	0.274	0.464	n.d.
	Snov_1360		0.918	0.860	0.613	0.942	n.d.	n.d.	0.638
	Snov_2769		n.d.	n.d.	n.d.	n.d.	n.d.	n.d.	n.d.
	Snov_0279		0.244	0.379	0.407	0.228	0.484	0.477	0.639
PEP carboxylase	Snov_2431	4.1.1.31	0.092	0.040	0.083	0.070	0.065	0.087	0.074
**Serine Pathway**									
glycine hydroxymethyl transferase	Snov_0422	2.1.2.1	0.510	0.484	0.533	0.531	0.492	0.544	0.994
	Snov_1652		0.107	0.119	0.101	0.236	0.105	0.114	0.135
serine: glyoxylate aminotransferase	Snov_2530	2.6.1.45	n.d.	0.832	0.497	0.643	n.d.	0.788	n.d.
hydroxypyruvate reductase	Snov_3108	1.1.1.81	0.068	0.072	0.093	0.117	0.158	0.095	0.078
glycerate kinase	Snov_3108	2.7.1.65	0.068	0.072	0.093	0.117	0.158	0.095	0.078
enolase	Snov_1800	4.2.1.11	0.029	0.057	0.029	0.028	0.028	0.026	0.029
phosphoglycerate mutase	Snov_0264	5.4.2.1	0.454	0.365	0.489	0.310	0.659	0.771	0.691
	Snov_0324		0.099	0.070	0.082	0.072	0.067	0.070	0.062
PEP carboxylase	Snov_2431	4.1.1.31	0.092	0.040	0.083	0.070	0.065	0.087	0.074
malate/lactate dehydrogenase	Snov_4339	1.1.1.37	n.d.	0.817	0.930	n.d.	0.754	1.089	n.d.
	Snov_3299		0.025	0.117	0.087	0.117	0.053	0.117	0.069
	Snov_1738		n.d.	n.d.	n.d.	n.d.	n.d.	n.d.	n.d.
	Snov_0198		1.014	0.834	0.740	0.932	1.000	0.891	0.762
	Snov_0154		0.563	0.627	0.427	0.673	0.386	0.587	0.341
malate thiokinase	Snov_2988	6.2.1.9	0.012	0.028	0.026	0.018	0.024	0.045	0.034
