# Supplementary material for: Metabolic adaptation and trophic strategies of soil bacteria—C1- metabolism and sulfur chemolithotrophy in Starkeya novella
Source: Front Microbiol. 2013 Oct 17;4:304. doi: 10.3389/fmicb.2013.00304 (PMC3797975; doi:10.3389/fmicb.2013.00304)
Supplement: Supplementary file 5 [file DataSheet4.PDF]

	gene locus	EC number	Glc	Glc/TS	Fruc	Fruc/TS	MeOH	MeOH/TS	TS
total proteins detected in the sample			2725	2790	2820	2829	2420	2333	2175
data are reported in percent as the relative position of the proteins in the list of proteins detected, with 1.0 or 100% indicating the lowest ranking									
**Complex I**									
**NADH: ubiquinol dehydrogenase**	Snov_1849	1.6.5.3	0.546	0.679	0.716	0.485	0.511	0.602	n.d.
	Snov_1850		0.488	0.529	0.548	0.600	n.d.	0.853	0.843
	Snov_1851		0.495	0.575	0.543	0.501	0.618	0.647	0.806
	Snov_1852		0.837	0.901	0.747	0.869	0.548	0.690	0.775
	Snov_1853		n.d.	n.d.	n.d.	n.d.	n.d.	n.d.	n.d.
	Snov_1854		1.040	0.794	1.022	0.956	1.080	0.916	n.d.
	Snov_1855		0.668	0.548	0.571	0.964	0.639	0.790	0.650
	Snov_1856		n.d.	n.d.	n.d.	0.452	n.d.	n.d.	n.d.
	Snov_1857		n.d.	0.977	0.951	1.019	0.945	n.d.	n.d.
	Snov_1858		0.039	0.036	0.045	0.039	0.023	0.045	0.042
	Snov_1859		0.085	0.116	0.125	0.118	0.244	0.265	0.316
	Snov_1860		0.218	0.195	0.091	0.164	0.218	0.240	0.269
	Snov_1861		0.408	0.436	0.560	0.435	0.419	0.641	0.643
	Snov_1862		0.302	0.244	0.170	0.200	0.208	0.149	0.489
	Snov_1863		0.401	0.529	0.715	0.527	0.481	0.356	0.531
	Snov_1864		0.982	0.984	0.802	0.810	0.593	0.591	0.845
**Complex II**									
**succinate DH flavosubunit**	Snov_3318	1.3.5.1	0.066	0.052	0.039	0.041	0.102	0.091	0.095
b556 SU	Snov_3320		n.d.	n.d.	n.d.	n.d.	n.d.	n.d.	n.d.
Fe/S SU	Snov_3317		0.135	0.322	0.246	0.221	0.312	0.349	0.467
**Complex III**									
cyt b/c1 complex	Snov_2477	1.10.2.2	0.195	0.124	0.164	0.109	0.505	0.180	0.448
	Snov_2478		0.253	0.123	0.151	0.292	0.428	0.295	0.196
	Snov_2479		0.259	0.281	0.392	0.445	0.463	0.384	0.522
**Complex IV**									
**cytochrome oxidases**									
aa3	Snov_0584	1.9.3.1	0.308	0.270	0.206	0.171	0.204	0.071	0.251
	Snov_0585		n.d.	n.d.	n.d.	0.397	n.d.	n.d.	n.d.
	Snov_0586		0.992	n.d.	0.989	n.d.	n.d.	n.d.	n.d.
	Snos_0589		n.d.	n.d.	n.d.	n.d.	n.d.	n.d.	n.d.
aa3/cyo	Snov_1015		n.d.	n.d.	n.d.	n.d.	n.d.	n.d.	n.d.
	Snov_1016		n.d.	n.d.	n.d.	n.d.	n.d.	n.d.	n.d.
	Snov_1019		n.d.	n.d.	n.d.	n.d.	n.d.	1.156	n.d.
aa3/cyo	Snov_4240		n.d.	n.d.	n.d.	n.d.	n.d.	n.d.	n.d.
	Snov_4241		0.905	n.d.	0.777	1.049	n.d.	n.d.	n.d.
	Snov_4242		n.d.	n.d.	n.d.	n.d.	n.d.	n.d.	n.d.
	Snov_4243		n.d.	n.d.	n.d.	n.d.	n.d.	n.d.	n.d.
cbb3 oxidase	Snov_4464		n.d.	n.d.	n.d.	0.783	0.931	0.946	n.d.
	Snov_4465		n.d.	n.d.	n.d.	0.665	0.931	n.d.	0.808
	Snov_4466		n.d.	n.d.	n.d.	0.428	0.724	n.d.	n.d.
	Snov_4467		n.d.	n.d.	n.d.	0.336	0.450	1.131	0.855
	Snov_4468		0.581	0.421	0.318	n.d.	n.d.	0.378	0.402
**quinol oxidases, bd type**									
Subunit I	Snov_620	1.10.3.-	n.d.	0.772	0.870	0.574	n.d.	1.173	n.d.
Subunit II (cytd)	Snov_0619		n.d.	n.d.	n.d.	n.d.	n.d.	n.d.	n.d.
Subunit I	Snov_3535		n.d.	n.d.	n.d.	n.d.	n.d.	n.d.	n.d.
Subunit II (cyt d)	Snov_3536		n.d.	n.d.	n.d.	n.d.	n.d.	n.d.	n.d.
**ATP-synthase**									
	Snov_4430	3.6.3.14	n.d.	n.d.	n.d.	n.d.	n.d.	n.d.	n.d.
	Snov_4431		n.d.	0.224	0.188	0.197	n.d.	0.441	n.d.
	Snov_4432		0.291	0.249	0.308	0.322	0.344	0.159	0.309
	Snov_4433		0.343	0.325	0.351	0.237	0.264	0.155	0.210
pyrophosphatase	Snov_2836	3.6.1.1.	0.140	0.280	0.210	0.165	0.195	0.290	0.206
polyphosphate kinase	Snov_1116		0.305	0.435	0.252	0.373	0.366	0.826	0.465
