# Supplementary material for: Metabolic adaptation and trophic strategies of soil bacteria—C1- metabolism and sulfur chemolithotrophy in Starkeya novella
Source: Front Microbiol. 2013 Oct 17;4:304. doi: 10.3389/fmicb.2013.00304 (PMC3797975; doi:10.3389/fmicb.2013.00304)
Supplement: Supplementary file 6 [file DataSheet5.PDF]

	gene locus	Glc	Glc/TS	Fruc	Fruc/TS	MeOH	MeOH/TS	TS
total proteins detected in the sample		2725	2790	2820	2829	2420	2333	2175
data are reported in percent as the relative position of the proteins in the list of proteins detected, with 1.0 or 100% indicating the lowest ranking								
**mxaF gene cluster**								
F	Snov_4188	0.0004	0.0004	0.004	0.007	0.0004	0.001	0.002
J	Snov_4189	0.055	0.059	0.108	0.080	0.010	0.024	0.050
G	Snov_4190	0.261	0.335	0.706	0.351	0.114	0.088	0.257
I	Snov_4191	0.124	0.276	0.195	0.462	0.227	0.171	0.354
R	Snov_4192	0.183	0.134	0.228	0.375	0.049	0.045	0.261
S	Snov_4193	1.045	0.822	n.d.	n.d.	0.372	0.496	0.982
A	Snov_4194	0.895	0.467	0.817	0.542	0.538	0.498	1.025
C	Snov_4195	0.479	0.820	0.473	0.916	0.704	0.724	n.d.
K	Snov_4196	0.505	0.454	0.805	0.602	0.215	0.213	0.580
L	Snov_4197	0.760	0.751	0.273	0.790	0.483	0.681	0.491
D	Snov_4198	0.146	0.298	0.765	0.448	0.194	0.261	0.349
E	Snov_4199	0.418	0.250	n.d.	0.317	0.109	0.127	0.134
H	Snov_4187	n.d.	n.d.	0.639	0.692	0.715	0.734	0.536
B	Snov_4185	0.341	n.d.	0.330	0.258	0.176	0.152	0.380
**xoxF gene cluster**								
F	Snov_1035	0.114	0.056	0.071	0.058	0.029	0.043	0.032
G	Snov_1036	0.457	0.408	0.412	0.545	0.178	0.197	0.413
J	Snov_1037	0.583	0.395	0.551	0.648	0.231	0.297	0.587
rhodanese	Snov_1038	0.815	0.516	0.953	n.d.	0.743	0.891	0.753
**formaldehyde activiating enzymes**								
	Snov_0740	0.377	0.402	0.316	0.228	0.452	0.576	0.817
	Snov_1050	0.052	0.073	0.099	0.051	0.008	0.018	0.021
**GSH dependent formaldehyde activating enzymes**								
fag1	Snov_1125	0.583	0.831	0.953	0.881	1.017	0.655	n.d.
fag2	Snov_1340	0.636	0.685	0.592	0.755	n.d.	n.d.	0.776
**Formate dehydrogenases**								
fdh1	Snov_3504	0.317	0.066	0.049	0.287	0.402	0.010	0.141
fdh2	Snov_3851	0.361	0.648	0.417	0.838	0.013	0.053	n.d.
